# Supplementary figures and images for: RNase-mediated reprogramming of Yersinia virulence
Source: PLoS Pathog. 2024 Aug 19;20(8):e1011965. doi: 10.1371/journal.ppat.1011965 (PMC11361751; doi:10.1371/journal.ppat.1011965)

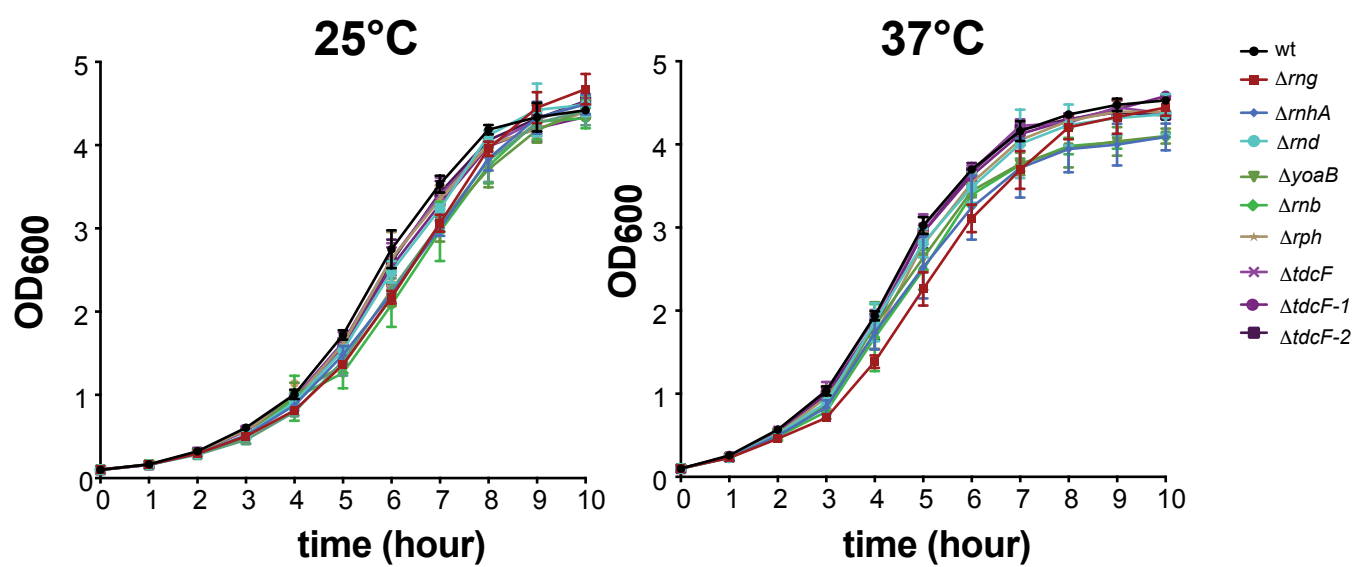

**Fig. S1:** Meyer *et al.* 2024

Supplement: S1 Fig — Overnight cultures of Y. pseudotuberculosis YPIII (wt) and the isogenic RNase mutants indicated on the right were diluted 1:50 in LB and growth at 25°C and 37°C was followed by measurement of OD600. Data represent the mean ± SD from experiments performed in triplicates. (PDF) [file ppat.1011965.s001.pdf]

**A**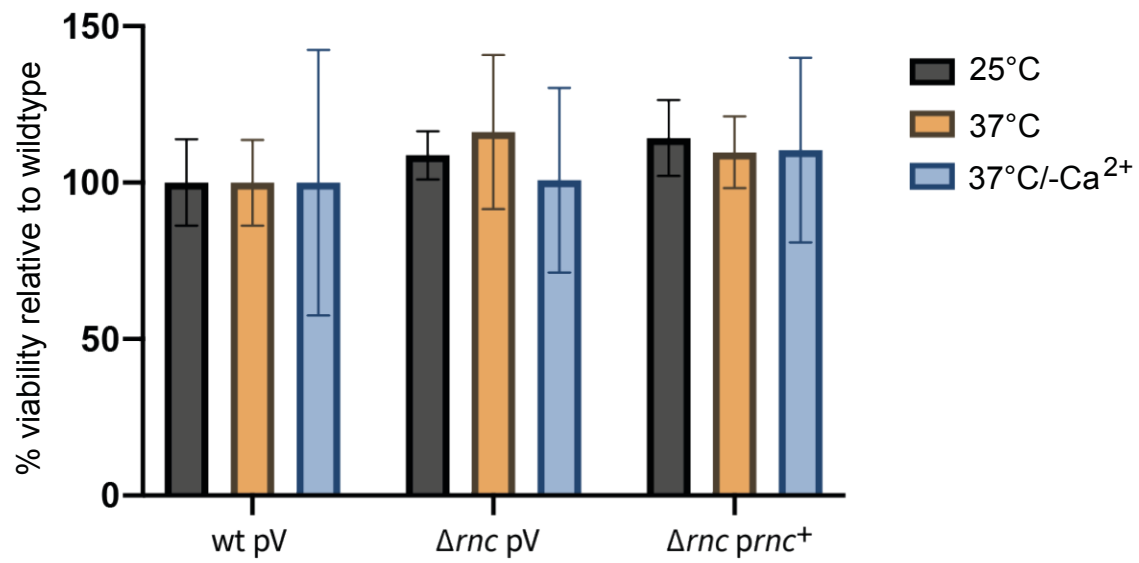**B**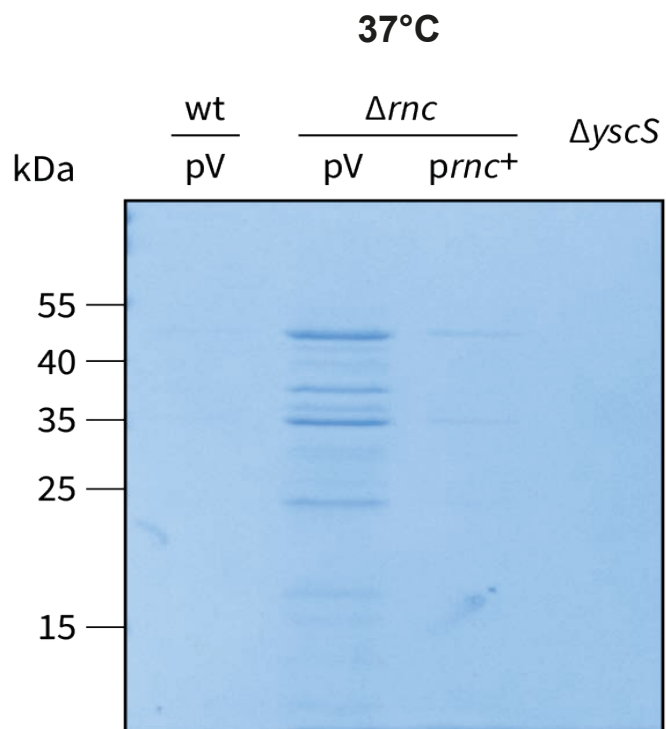

**Fig. S2** Meyer *et al.* 2024

Supplement: S2 Fig — (A) Viability of Y. pseudotuberculosis strains YPIII (wt), YP356 (Δrnc), and YP356 (Δrnc) pIVO20 (prnc+). Data represent the mean ± SD from four independent biological replicates (lower panel). No significant differences compared to the wildtype were determined using the Student’s t-test. (B) Y. pseudotuberculosis strains YPIII (wt), YP356 (Δrnc), and YP356 (Δrnc) pIVO20 (prnc+) were grown at 37°C for 4 h; the secreted proteins in the supernatant of the cultures were precipitated with TCA and separated on SDS gels. The Yop secretion-deficient mutant YP101 (ΔyscS) was used as a negative control. (PDF) [file ppat.1011965.s002.pdf]

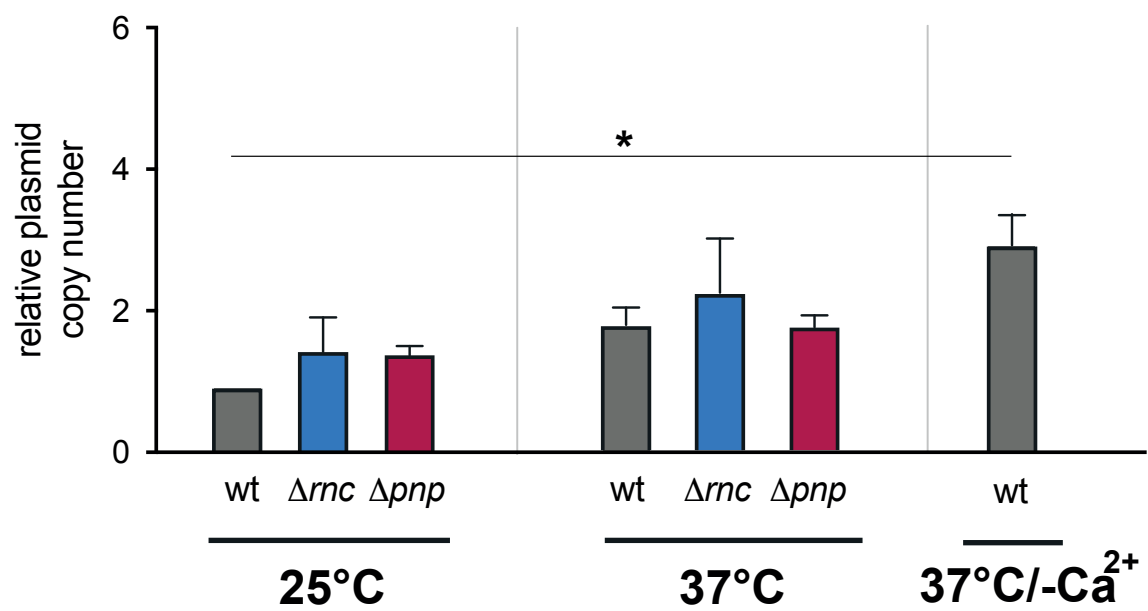

Fig. S3: Meyer *et al.* 2024

Supplement: S3 Fig — Y. pseudotuberculosis strains YPIII (wt), YP139 (Δpnp), and YP356 (Δrnc) were grown at 25°C, and 37°C, and the wildtype YPIII also at 37°C/-Ca2+, i.e. T3SS/Yop-inducing conditions. The total DNA of the strains grown under the different conditions was prepared and the copy number of the virulence plasmid was determined by qPCR. The relative plasmid copy number of wildtype was compared with the different mutant strains. Data represent the mean ± SD from three independent biological replicates. Significant differences were determined using the Student’s t-test and are indicated by asterisks (* P<0.05). (PDF) [file ppat.1011965.s003.pdf]

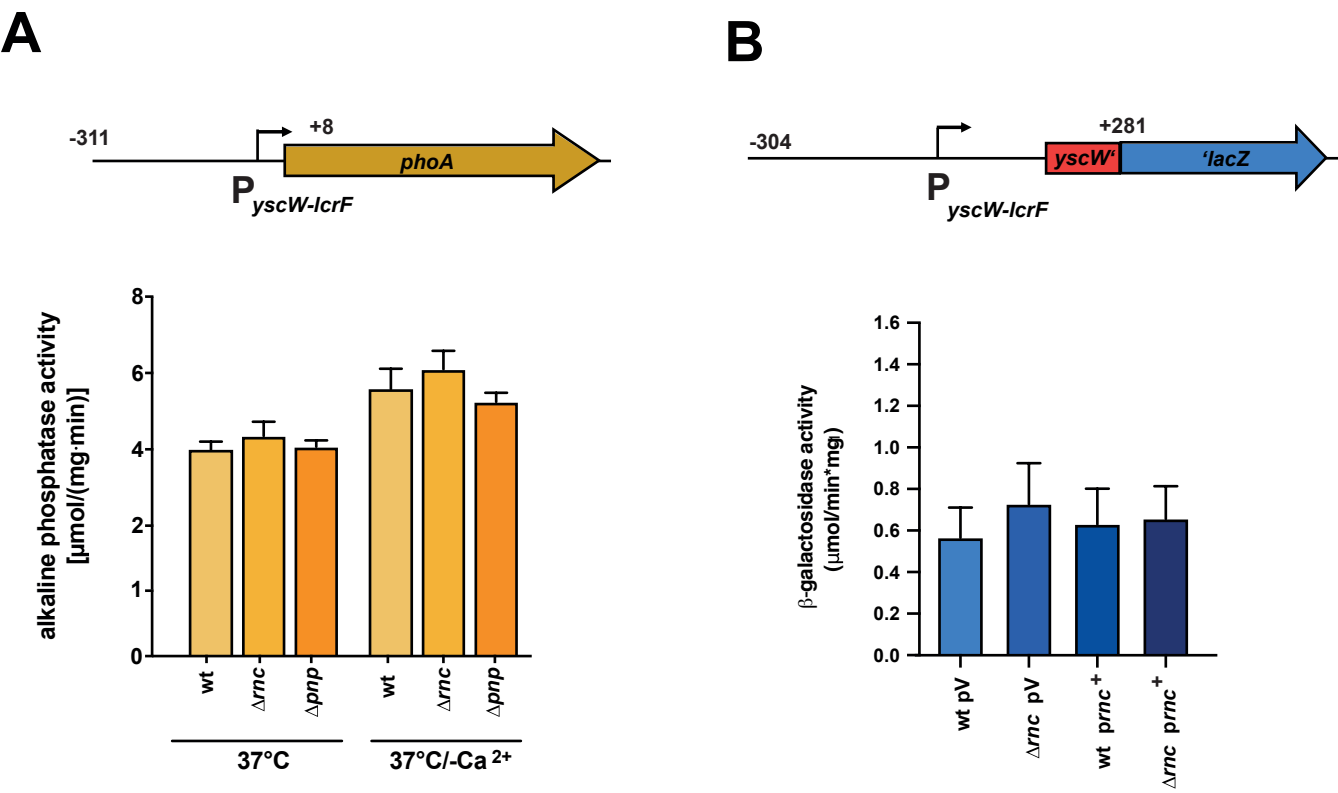

Fig. S4: Meyer *et al.* 2024

Supplement: S4 Fig — (A) Plasmid pMV53 encoding the yscW-phoA transcriptional fusion and (B) plasmid pKB35 encoding the yscW-lacZ transcriptional fusion were transformed into Y. pseudotuberculosis strains YPIII (wt) pAKH85 (pV, empty vector), YP356 (Δrnc) pAKH85 (pV, empty vector) and the complementing strains YPIII (wt) pIVO20 (prnc+) and YP356 pIVO20 (prnc+). The transformants were grown at 37°C and alkaline phosphatase or beta-galactosidase activity was determined, respectively. Data represent the mean ± SD from three independent biological replicates. Significant differences were determined using Student’s t-test and indicated by asterisks (*P <0.05). (PDF) [file ppat.1011965.s004.pdf]

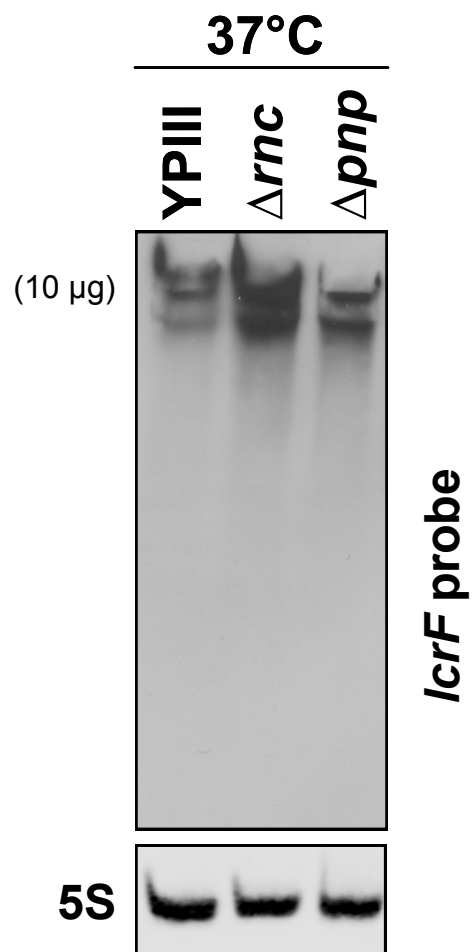

Fig. S5: Meyer *et al.* 2024

Supplement: S5 Fig — Y. pseudotuberculosis (YPIII), YP356 (Δrnc), and YP139 (Δpnp) were grown at 37°C, and the total RNA of the strains was prepared. 10 μg of total RNA of the different strains were loaded onto a denaturing acrylamide gel to allow the detection of smaller degradation products. The lcrF transcripts were identified by Northern blot using a probe covering the lcrF coding sequence. The blot represents one of two biological replicates. The 5 S rRNA was used as loading control. (PDF) [file ppat.1011965.s005.pdf]

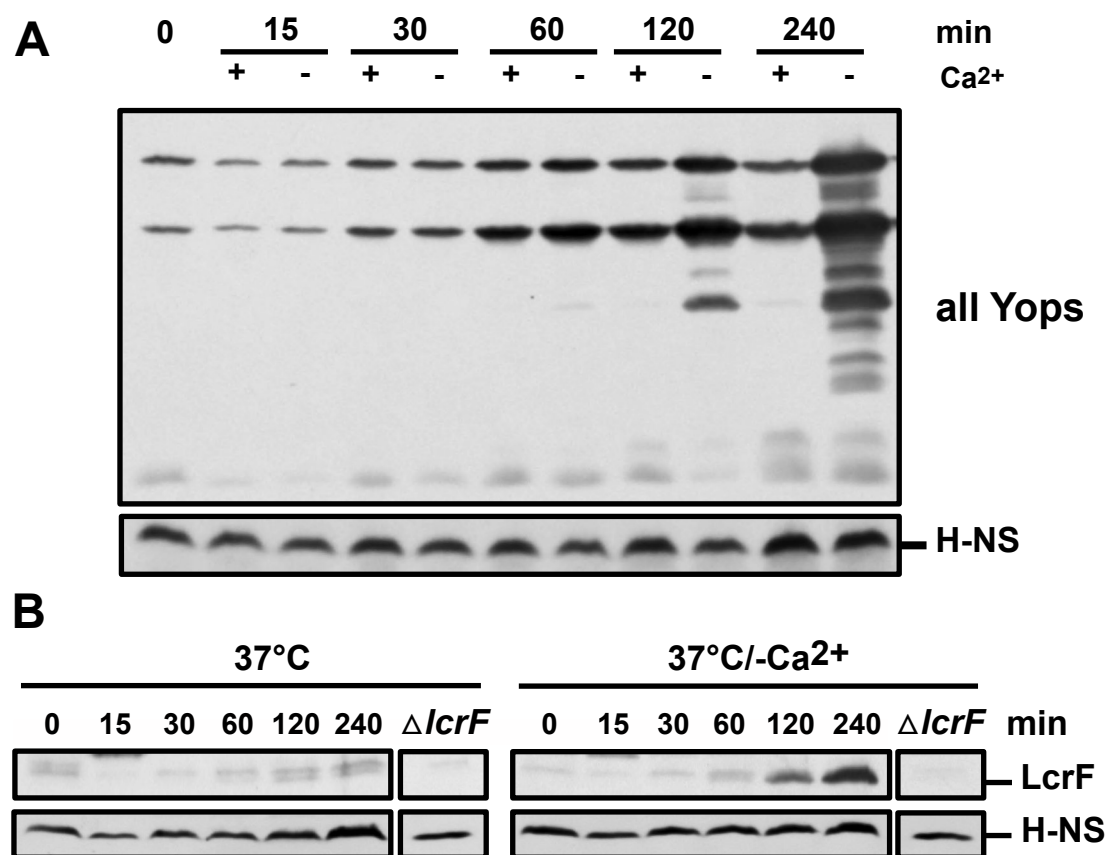

Fig. S6 Meyer *et al.* 2024

Supplement: S6 Fig — Y. pseudotuberculosis (YPIII) was grown over day at 25°C to exponential phase and was then shifted to 37°C in the presence (37°C) or absence of Ca2+ (-Ca2+). Whole-cell extracts were prepared and the amounts of Yops (A) and LcrF (B) were analyzed by Western blotting using all-Yop and LcrF polyclonal antisera. H-NS was used as loading control. The ΔlcrF mutant was used as a negative control. (PDF) [file ppat.1011965.s006.pdf]

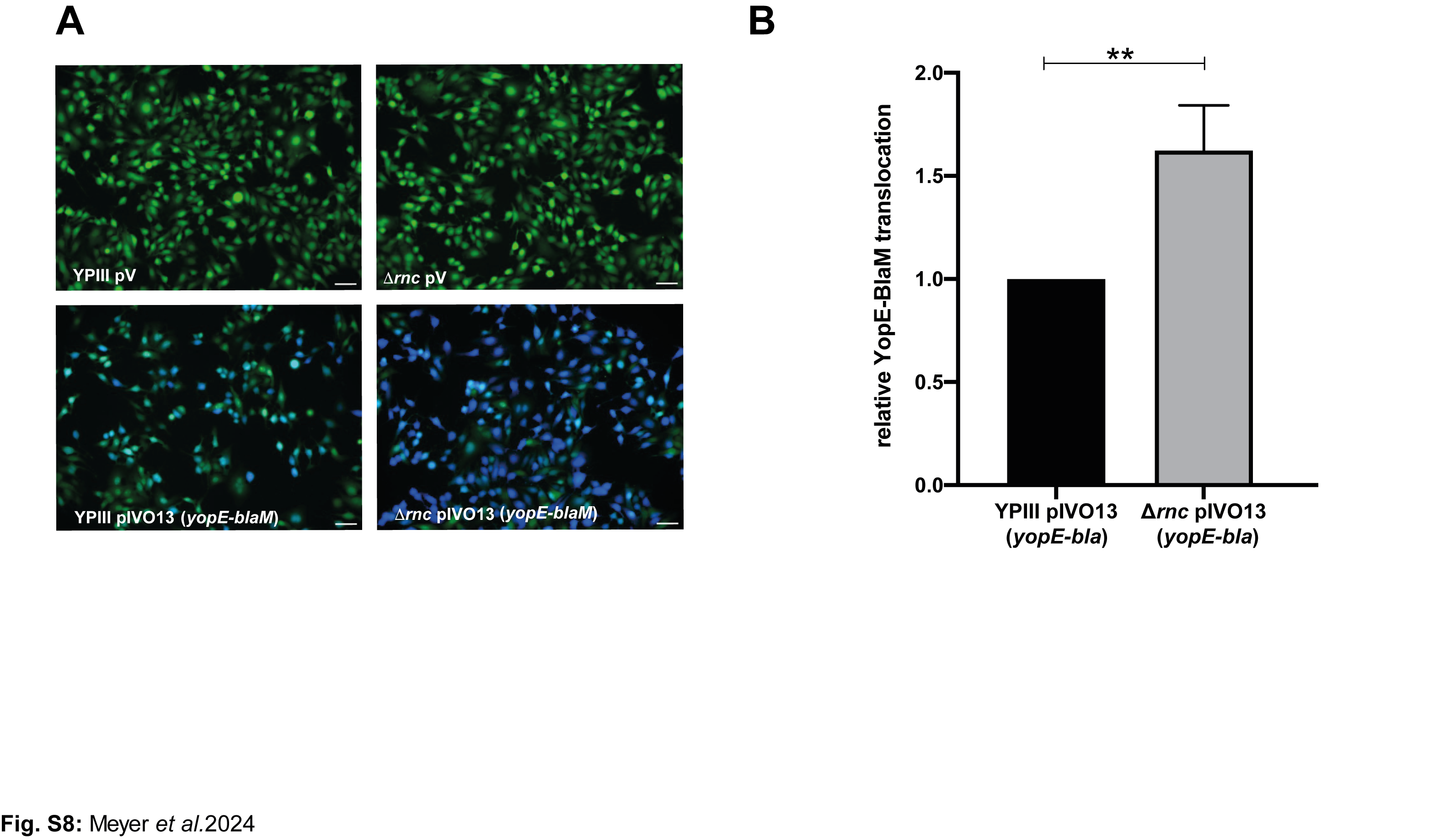

Supplement: S8 Fig — (A) Translocation of a plasmid pIVO13-encoded YopE-beta-lactamase (BlaM) fusion protein expressed in Y. pseudotuberculosis strains YPIII (wt), and YP356 (Δrnc) into human epithelial cells (HEp-2) was determined by a green to blue fluorescence shift. Bacteria transformed with the empty plasmid (pV) were used as negative control. White bar: 50 μm. (B) Relative translocation of the YopE-BlaM construct determined by the ratio of blue to green fluorescent cells is illustrated. Data represent the mean ± SD from three independent biological replicates relative to the YopE-BlaM translocation determined for the wildtype defined as 1.0. Significant differences were determined using the Student’s t-test and are indicated by asterisks (*** P<0.001). (TIF) [file ppat.1011965.s008.tif]
