# Supplementary material for: RNase-mediated reprogramming of Yersinia virulence
Source: PLoS Pathog. 2024 Aug 19;20(8):e1011965. doi: 10.1371/journal.ppat.1011965 (PMC11361751; doi:10.1371/journal.ppat.1011965)
Supplement: S7 Fig — (A) Two-dimensional biplot and (B) three-dimensional principal component analysis of mean-centered and scaled rlog-transformed read count values of the Y. pseudotuberculosis wildtype strain YPIII and the isogenic Δrnc mutant grown over day at 25°C to exponential phase (T0) and then shifted to 37°C in the presence or absence of Ca2+ for 1 h (T1) or 4 h (T2). (PDF) [file ppat.1011965.s007.pdf]

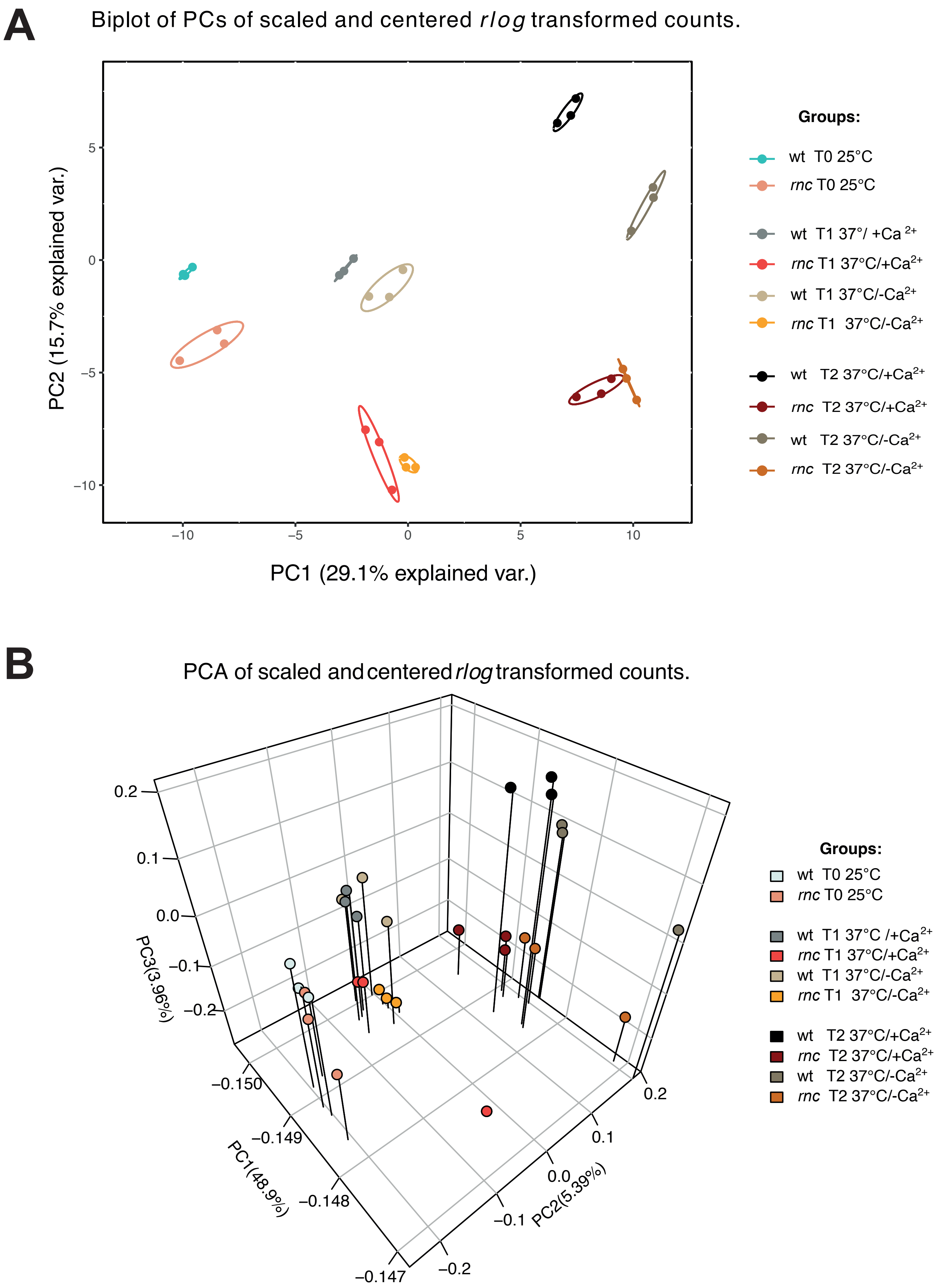

Fig. S7: Meyer *et al.* 2024
