# Supplementary material for: RNase-mediated reprogramming of Yersinia virulence
Source: PLoS Pathog. 2024 Aug 19;20(8):e1011965. doi: 10.1371/journal.ppat.1011965 (PMC11361751; doi:10.1371/journal.ppat.1011965)
Supplement: S1 Table — The transcription profile obtained by an RNA-seq analysis of Y. pseudotuberculosis wildtype strain YPIII and its isogenic Δrnc mutant grown at 37°C were compared. Transcripts of ribosomal proteins and other translation-relevant factors that were found in a significantly higher or lower abundance in the Δrnc mutant (log2-fold change (log2FC) ≥ +/- 2, p-value ≤ 0.05) are listed. (PDF) [file ppat.1011965.s009.pdf]

Table S1: RNase III-dependent mRNA changes of translation-relevant factors

Ribosomal transcripts

Gene id	Gene	YPIII 37°C vs. Δ*rnc* 37°C $\log_2$ FC	p-value
YPK_0275	*rpsL*	1.097	0.04
YPK_0282	*rpsJ*	3.059	0.00
YPK_0283	*rplC*	2.784	0.00
YPK_0284	*rplD*	1.419	0.04
YPK_0298	*rplF*	1.411	0.05
YPK_0335	*rplK*	1.973	0.00
YPK_1822	*rpmI*	1.782	0.01
YPK_1823	*rplT*	1.572	0.05
YPK_3211	*rpmJ-2*	1.059	0.01
YPK_3210	*rpmE-2*	-1.278	0.00
YPK_3781	*rplI*	-1.015	0.05

Translation-relevant factors

Gene id	Gene	YPIII 37°C vs. Δ*rnc* 37°C $\log_2$ FC	p-value
YPK_1821	*infC*	1.015	0.04
YPK_3318	*mtnA*	-1.394	0.00
YPK_3363	*rimM*	1.837	0.00
YPK_4218	*rsmG/gidB*	3.388	0.00
YPK_3353	*raiA/yfiA*	-1.414	0.00
