# Supplementary material for: RNase-mediated reprogramming of Yersinia virulence
Source: PLoS Pathog. 2024 Aug 19;20(8):e1011965. doi: 10.1371/journal.ppat.1011965 (PMC11361751; doi:10.1371/journal.ppat.1011965)
Supplement: S2 Table — Upper panel: Illustration of the localization of the Ysc-T3SS/Yop components forming the injectisome of Yersinia upon host cell contact. Lower panel: The transcription profile obtained by an RNA-seq analysis of Y. pseudotuberculosis wildtype strain YPIII and its isogenic Δrnc mutant grown at 37°C were compared, and transcripts of genes encoding the Ysc-T3SS/Yop secretion machinery that were found in a significantly higher or lower abundance in the Δrnc mutant (log2-fold change (log2FC) ≥ +/- 2, p-value ≤ 0.05) are listed, and their functions are indicated on the right. (PDF) [file ppat.1011965.s010.pdf]

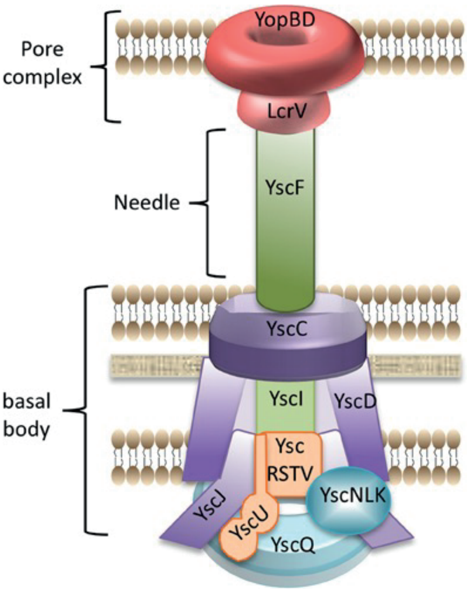

		T1			T2			
**Gene Symbol**	**Genbank Acc**	wt 37°C<br>wt -Ca2+	wt 37°C<br>△*rnc* 37°C	wt -Ca2+<br>△*rnc* -Ca2+	wt 37°C<br>wt -Ca2+	wt 37°C<br>△*rnc* 37°C	wt -Ca2+<br>△*rnc* -Ca2+	
virG, yscW	pYV0075	1.561	3.214	2.293	3.144	4.475	1.983	
lcrF, virF	pYV0076	0.903	2.879	2.060	2.807	3.011	0.623	
yscM, lcrQ	pYV0089	1.287	1.548	0.804	2.844	2.033	-0.579	
yopN, lcrE	pYV0065	1.740	1.437	0.174	2.377	2.183	-0.170	
tyeA	pYV0064	2.651	2.128	0.463	2.935	2.979	0.661	
yscP	pYV0069	-0.437	0.061	-0.350	-0.099	-0.116	0.057	**Regulation**
yscU	pYV0074	0.488	0.750	0.494	1.591	2.073	1.251	
lcrR	pYV0059		0.813	0.129	1.516	1.167	-0.199	
lcrG	pYV0058		0.917	1.407	4.472	3.324	-0.204	
yscH, yopR	pYV0084		1.677	0.618	0.765	1.630	0.787	
yscA	pYV0077	0.856	7.115	5.764	1.588	7.341	6.038	
yopK, yopQ	pYV0040	3.301	4.449	2.526	7.530	7.220	0.966	
yopT	pYV0041		1.472	3.409	4.443	5.285	1.592	
yopP, yopJ	pYV0098	2.408	3.175	2.199	5.809	5.641	1.147	
ypkA/yopO	pYV0001	0.483	0.855	0.813	1.647	1.206	0.073	**Secreted effectors. translocators**
yopM	pYV0047	1.182	2.441	2.063	5.000	5.084	1.049	
yopE	pYV0025	-0.284	-0.225	-0.575	1.391	0.392	-0.615	
yopH	pYV0094	2.118	1.972	1.067	4.587	4.639	0.337	
yopD	pYV0054	2.697	2.819	1.668	5.544	5.104	-0.382	
yopB	pYV0055	1.625	1.895	1.641	4.259	4.070	-0.343	
lcrV	pYV0057	2.264	4.205	2.476	4.566	5.679	1.704	
yscF	pYV0082		2.102	0.969	1.232	2.230	1.241	
yscC	pYV0079	0.197	0.706	0.328	0.250	0.909	1.100	
yscD	pYV0080	0.321	1.106	1.193	0.773	1.229	0.261	**Basal body**
yscJ, ylpB	pYV0086	0.995	0.939	1.153	2.261	2.140	0.014	
yscI, lcrO	pYV0085	1.199	1.169	0.473	1.213	0.918	-0.241	
yscR	pYV0071	-0.348	0.332	0.307	0.004	0.263	0.771	
yscS	pYV0072		1.089	0.892	1.066	1.369	0.988	
yscT	pYV0073		0.621	0.943	1.413	1.063	-0.132	**Export apparatus**
lcrD, yscV	pYV0060	1.150	1.837	1.239	1.899	4.131	2.887	
yscU	pYV0074	0.488	0.750	0.494	1.591	2.073	1.251	
yscQ	pYV0070	-0.638	-0.835	-1.395	-0.441	-0.302	-0.228	
sctN/yscN	pYV0067	1.853	1.952	0.825	2.056	1.757	0.350	
yscL	pYV0088	1.239	5.204	4.331	4.510	7.763	3.383	**Sorting platform**
yscK	pYV0087	-0.178	0.186	-0.483	-0.214	-0.931	-0.197	
yscY	pYV0061		-0.200	-0.319	-0.285	-0.227	0.143	
yscX	pYV0062		-0.336	-0.587	-0.848	-0.893	-1.387	
yscO	pYV0068		1.044	0.517	2.775	2.365	-0.129	
yscE	pYV0081		0.863	-0.266	-1.004	-0.953	0.118	
yscG	pYV0083		2.187	0.630	2.486	2.588	-0.231	

Table S2
