# Supplementary material for: RNase-mediated reprogramming of Yersinia virulence
Source: PLoS Pathog. 2024 Aug 19;20(8):e1011965. doi: 10.1371/journal.ppat.1011965 (PMC11361751; doi:10.1371/journal.ppat.1011965)
Supplement: S3 Table — All bacterial strains and plasmids constructed and used in this study are listed and described, and their source and reference are indicated. (PDF) [file ppat.1011965.s011.pdf]

Table S3: Bacterial strains and plasmids

Strains, plasmids	Description	Source and reference
**Bacterial strains** *E. coli* **K12**		
BL21λDE3	F$^-$ *ompT gal dcm lon hsdSB*(rB$^-$ mB$^-$) λDE3	[1]
CC118λ*pir*	F$^-$ Δ(*ara-leu*)7697 Δ(*lacZ*)74 Δ(*phoA*)20 *araD139 galE galK thi rpsE rpoB arfE*^{am} *recA1*, λ*pir*	[2]
S17-1λ*pir*	*recA thi pro hsdR*$^-$ M1$^+$ (RP4-2Tc::Mu-Km::Tn7), λ*pir*	[3]
DH5α	F$^-$Φ80*lacZ*ΔM15 Δ(*lacZYA-arg*F) U169 *recA1endA1hsdR17* (rk-, mk+) *phoAsupE*44λ - *thi*-1 *gyrA*96 *relA1*	Invitrogen
Y. pseudotuberculosis		
YPIII	pIB, wildtype	[4]
YP45	YPIII, Δ*flhDC,* Kn^R	[5]
YP53	YPIII, Δ*csrA*, Kan^R	[5]
YP69	YPIII, Δ*csrB*	[5]
YP80	YPIII, Δ*hfq*	[6]
YP89	YPIII, Δ*crp*	[7]
YP91	YPIII, Δ*yopD*	[8]
YP101	YPIII, pYV0072 Δ*yscS*	R. Steinmann
YP126	YPIII, Δ*csrC*	[5]
YP139	YPIII, Δ*pnp*; Kn^R	[8]
YP145	YPIII, Δ*yopD*, Δ*csrA*; Kn^R	[8]
YP179	YPIII, Δ*lcrF*	[8]
YP218	YPIII, Δ*yopD, Δpnp*	[8]
YP356	YPIII, Δ*rnc*	This study
YP372	YPIII, pYV0054 Δ*yopD*, Δ*rnc*	This study
YP375	YPIII, Δ*pnp,* Δ*rnc*; Kn^R	This study
**Plasmids**		
pFU86	pFU53 *phoA* + RBS, Amp^R	[9]
pAKH3	pGP704, *sacB*$^+$, Ap^R	[8]
pAKH85	pACYC184, Δ*tet*, Cm^R	[10]
pISN1	pFU53, ori ColE1, *yopE*-TEM , Cm^R	Isabell Nitzel
pIVO11	pAKH3, Δ*rnc*, Cb^R	This study
pIVO13	pPCH1, *yopE-blaM*	This study
pIVO20	pAKH85, P_{rnc}, 5'UTR *rnc*, *rnc*$^+$, Cm^R	This study
pIVO21	pAKH85, P_{pnp}, 5'UTR *pnp*, *pnp*$^+$, Cm^R	This study
pKB34	pTS02, *yscW*(-573)^b*lcrF*`-`*lacZ*(25)^d, Amp^R	[8]
pKB35	pTS02, *yscW*(-573)^b`-`*lacZ*(5)^d, Amp^R	[8]
pMV53	pTT15 *yscW*(-575 to -256)^b-*phoA*	This study
pPCH1	pISN1, ori CoE1, *yopE*-TEM, Cm^R	Paweena Chaoprasid
pTS02	pGP20, ori pSC101, *lacZ*, Amp^R	[11]
pTS03	pGP20, ori pSC101, RBS-*lacZ*$^+$, Amp^R	This study
pTT15	pFU86-P_{tet}::MCS-*phoA*	This study

[a] the number indicates the nucleotide relative to the transcriptional start site

[b] the number indicates the nucleotide relative to the translational start site

[c] the number indicates the codon of the corresponding gene fused to the reporter gene

References:

1. Studier FW, Moffatt BA. Use of bacteriophage T7 RNA polymerase to direct selective high-level expression of cloned genes. Journal of molecular biology. 1986;189: 113–30.
2. Manoil C, Beckwith J. A genetic approach to analyzing membrane protein topology. Science (New York, NY). 1986;233: 1403–8.
3. Simon R, Priefer U, Pühler A. A Broad Host Range Mobilization System for In Vivo Genetic Engineering: Transposon Mutagenesis in Gram Negative Bacteria. Bio/Technology. 1983;1: 784–791. doi:10.1038/nbt1183-784
4. Bolin I, Norlander I, Wolf-Watz H, I Bölin LN. Temperature-inducible outer membrane protein of Yersinia pseudotuberculosis and Yersinia enterocolitica is associated with the virulence plasmid. Infect Immun. 1982;37: 506-512.
5. Heroven AK, Bohme K, Rohde M, Dersch P. A Csr-type regulatory system, including small non-coding RNAs, regulates the global virulence regulator RovA of *Yersinia pseudotuberculosis* through RovM. Mol Microbiol. 2008;68: 1179–95. doi:MMI6218 [pii] 10.1111/j.1365-2958.2008.06218.x
6. Böhme K, Heroven AK, Lobedann S, Guo Y, Stolle A-S, Dersch P. The Small Protein YmoA Controls the Csr System and Adjusts Expression of Virulence-Relevant Traits of Yersinia pseudotuberculosis. Front Microbiol. 2021;12: 706934. doi:10.3389/fmicb.2021.706934
7. Nuss AM, Heroven AK, Waldmann B, Reinkensmeier J, Jarek M, Beckstette M, et al. Transcriptomic profiling of *Yersinia pseudotuberculosis* reveals reprogramming of the Crp regulon by temperature and uncovers Crp as a master regulator of small RNAs. PLoS Genet. 2015;11: e1005087. doi:10.1371/journal.pgen.1005087
8. Kusmierek M, Hoßmann J, Witte R, Opitz W, Vollmer I, Volk M, et al. A bacterial secreted translocator hijacks riboregulators to control type III secretion in response to host cell contact. PLoS Pathog. 2019;15: e1007813. doi:10.1371/journal.ppat.1007813
9. Uliczka F, Pisano F, Kochut A, Opitz W, Herbst K, Stolz T, et al. Monitoring of gene expression in bacteria during infections using an adaptable set of bioluminescent, fluorescent and colorigenic fusion vectors. PLoS One. 2011;6: e20425. doi:10.1371/journal.pone.0020425
10. Heroven AK, Dersch P. RovM, a novel LysR-type regulator of the virulence activator gene *rovA*, controls cell invasion, virulence and motility of *Yersinia pseudotuberculosis*. Mol Microbiol. 2006;62: 1469–83.
11. Böhme K, Steinmann R, Kortmann J, Seekircher S, Heroven AK, Berger E, et al. Concerted actions of a thermo-labile regulator and a unique intergenic RNA thermosensor control *Yersinia* virulence. PLoS Pathog. 2012;8: e1002518. doi:10.1371/journal.ppat.1002518
