# Supplementary material for: RNase-mediated reprogramming of Yersinia virulence
Source: PLoS Pathog. 2024 Aug 19;20(8):e1011965. doi: 10.1371/journal.ppat.1011965 (PMC11361751; doi:10.1371/journal.ppat.1011965)
Supplement: S4 Table — All nucleotides used in this study for cloning for mutant or plasmid constructions, qPCR, or the synthesis of Northern blot probes are indicated. (PDF) [file ppat.1011965.s012.pdf]

## Table S4: Oligonucleotides

| Primer | Sequence 5´- 3´ | Restriction site/Target |
|---|---|---|
| **Knockout YPK_1189 (Δ*rnc*)/pIVO11** | | |
| III981 | TGAACGGCAGGTATATGTG | |
| III982 | CACTTAACGGCTGACATGG | |
| VII879 | GCGGCGGAGCTCCCCTTCTCAGCTGAGATTGGC | *Sac*I |
| VII880 | CCAACAGCAGAACCAGTAATAGG | |
| VII881 | CCTATTACTGGTTCTGCTGTTGG | |
| VII882 | GCGGCGGAGCTCCGCTTCTGGGGTTTTGTACC | *Sac*I |
| VII366 | CAGTGCAGATAGCCGCTTC | |
| VII367 | CATATGCTTGCGCACAATGC | |
| **pIVO13** | | |
| VIII193 | GCGGCGCTCGAGTAGTATCTGGAATAGACAACGAAAGTTG | *yopE*, *Xho*I |
| VIII194 | GCGGCGGGATCCTTGCTGTGAGACTGAGCGC | *yopE*, *Bam*HI |
| I984 | TAAGAAACCATTATTATCATG | |
| V824 | CGTGCACCCAACTGATCT | |
| **Northern blot** | | |
| VIII255 | CATCCAGATTACCGACAAACC | *repA* |
| VIII256 | CCGAAGGCTTCACATCTCG | *repA* |
| VIII568 | GGAGTTCCTGGGTGACTC | *rnc* |
| VIII569 | GCTTGAGCCAGTACCTATTAC | *rnc* |
| 5'IDR700 CsrB | CAATGTTATACACCATCCCGGCGTTAATCC | *csrB* |
| 5'IDR700 CsrC | TTAGATAACGTCCCGGTTTCTCCCTCCTGC | *csrC* |
| 5'IDR700 5 S RNA | CTCTCGCATGGGGAGACCCCACACTACCATC | 5 S rRNA |
| IX855 | GGCATCACTAGAGATTATTAAATTAGAATGGG | *lcrF* |
| IX856 | GGGTGTGGAGTTGTCTCGTC | *lcrF* |
| IX857 | GACGAGACAACTCCACACCC | *lcrF* |
| IX858 | CCACCCTTGTAGATAATTTTCCTCC | *lcrF* |
| IX859 | GGAGGAAAATTATCTACAAGGGTGG | *lcrF* |
| IX860 | GCCTGTGGTTGCTATTTTAGTAAGAC | *lcrF* |
| **qPCR** | | |
| VII918 | AATTACCCAAGTGGGACACG | *yscM* |
| VII919 | TCACTATCACTTCCCCTGCCT | *yscM* |
| VII920 | TATCAAACTGAACGTAAGGCTC | *repA* |
| VII921 | AACGATAGCTTCAATGTCACG | *repA* |
| VII922 | GGCATGGATCCTGAGATGA | *glnA* |
| VII923 | CGTCAAAACATGTTCAGCGGAC | *glnA* |
| VII924 | AATCGTCAAGGTGTATCTGG | *rpoB* |
| VII925 | TCGTAAGGCATATCTTCGAT | *rpoB* |
| VII926 | ATCCACTGTTACCAGAATGC | YPK_3178 |
| VII927 | AGAAGAAACAACGGTCAAAA | YPK_3178 |
| **Complemen-tation plasmids** | | |
| II143 | GCACTGCGGCCGCTTATTTCAGCCCCAGAGC | pTT15 |
| VI527 | GGTCGGGGATCCTCCCTATCAGTGATAGAGATTGACATCCCTATCAGTGATAGAGA TACT GAGCACATC CGGGTACCTGCAGCTAGC | pTT15 |
| VIII385 | GTAGCACCTGAAGTCAGCC | pAKH85 |
| VIII388 | CCCTTATGCGACTCCTGC | pAKH85 |
| VIII365 | GCGCCAAGCTTCGTTGATGTTGCAGTTAGGC | pIVO20 |
| VIII387 | GCGCCGCATGCTCATTCAAGCTCCAACTGTTTC | pIVO20 |
| VIII636 | GCGCCAAGCTTGTCTGCGAGTTTCGGGAG | pIVO21 |
| VIII637 | GCGCCGGATCCTTACTCTGCTGCTGCTTCTG | pIVO21 |
| IX729 | GCGGCGGGATCCTAGGCTGCAATGTAACTAGG | pMV53 |
| IX730 | GCGGCGGGTACCGACAATACTGTATTATTTGTATTCAAC | pMV53 |
| ***lacZ*** | | |

| | | |
|---|---|---|
| **reporter plasmids** | | |
| II126 | GAGGGGACGACGACAGTATC | pTS02/03 |
| VI527 | GGTCGGGGATCCTCCCTATCAGTGATAGAGATTGACATCCCTATCAGTGATAGAGA TACTGAGCACATC CGGGTACCTGCAGCTAGC | pTT15 |
| II143 | GCACTGCGGCCGCTTATTTCAGCCCCAGAGC | pTT15 |
| IX729 | GCGGCGGGATCCTAGGCTGCAATGTAACTAGG | pMV53 |
| IX730 | GCGGCGGGTACCGACAATACTGTATTATTTGTATTCAAC | pMV53 |
